# Female students as victims of sexual abuse at institutions of higher learning: insights from Kwazulu-natal, South Africa

**DOI:** 10.1007/s43545-023-00611-z

**Published:** 2023-02-11

**Authors:** Mandisa Samukelisiwe Makhaye, Sazelo Michael Mkhize, Ephraim Kevin Sibanyoni

**Affiliations:** 1 Department of Criminal Justice, University of Zululand, Empangeni, South Africa; 2 Department of Criminology and Forensic Studies, University of KwaZulu-Natal, Durban, South Africa; 3 Department of Corrections Management, University of South Africa, Pretoria, South Africa

**Keywords:** University, Female student, Sexual victimisation, Underreporting

## Abstract

The focus of the wider study on which this paper is based was to uncover the social context of the sexual victimisation phenomenon in three selected institutions of higher learning in Durban, KwaZulu-Natal Province, South Africa. The objectives of the study were to examine the nature of sexual victimisation against female students in selected universities in Durban; to determine the factors that contribute to the sexual victimisation of female students; to investigate the effects of sexual victimisation on female students and to explore the reasons for female students’ reluctance to report sexual victimisation. The sample comprised a total of 60 participants  who had been purposively selected from three universities in the study area. The data were collected by means of interviews and were thematically analysed. The study found that incapacitated rape is more common on campuses than forcible rape, which is when perpetrators use threats and/or physical force. This could be attributed to the excessive use of alcohol and drugs by both victims and perpetrators. The study also confirmed that female students are reluctant to report sexual victimisation to the authorities. A key recommendation is that security measures need to be upgraded to ensure a safe environment for female students on campuses in the study area.

**Supplementary Information:**

The online version contains supplementary material available at 10.1007/s43545-023-00611-z.

## Introduction

Sexual violence amongst college and university students is a widespread problem that requires the urgent attention of academics, administrators, and policymakers (Perez-Trujillo et al. 2019). This paper thus responds to the urgent need to address and eradicate the sexual victimisation of female university students. The broader study has contributed to the existing body of knowledge by proving empirical data on sexual victimisation in South African institutions of higher learning, with particular emphasis on three universities in the Durban area. This paper proposes a working mechanism to curb the scourge of sexual victimisation in tertiary institutions as there are no such mechanisms in place, as confirmed by Dastile (2004). The authors agree as they noted a marked lack of studies on sexual victimisation in tertiary institutions in South Africa, whilst a plethora of literature exist on this phenomenon in international universities and colleges (Fisher et al. 2000; Jordan et al. 2014). International studies have documented the widespread nature of sexual victimisation of college women and the impact of this form of violence on female students, yet sexual victimisation in the South African tertiary context has been underreported. Most South African studies on this topic focused in general on crimes committed against female students (Mafadza 2020), but limited research focused on African female students, or Afrocentrism, particularly in KwaZulu-Natal. It is against this backdrop that this paper addresses the nature of sexual victimisation and its impact on female students in three selected universities in KwaZulu-Natal. It was argued that, although international studies have explored the sexual victimisation of female students and the trauma associated with it, there is a dire need to expose South African female students’ plight associated with sexual victimisation in universities in KwaZulu-Natal.

For the purpose of this paper, the term ‘university’ refers to a tertiary institution or an institution of higher learning.

### Sexual victimisation in south african higher education institutions

There are twenty-six (26) higher education institutions in South Africa with approximately two million students and staff across 420 campuses. Recent studies and statistics have confirmed that sexual assault is a major problem on these campuses, with 20 to 25% of women reporting sexual victimisation during their time at university. However, these figures are not a true reflection of the actual extent of this problem as female students seldom report sexual victimisation, and statistics of this crime may thus not be reliable. It is also a fact that universities do not keep such records or collect statistics of the sexual victimisation of students, whether male or female (Department of Higher Education and Training 2016). Fisher et al. (2000) estimate that a college with 10 000 female students might experience more than 350 rapes each year, but this cannot be confirmed as female victims are reportedly reluctant to report sexual victimisation.

As microcosms of the larger community, South African universities are equally prone to sexual violence. Women and girls are exposed to rape and other forms of gender-based violence on a “large and seemingly uncontrollable scale,” whilst the perpetrators are seldom brought before the law because female student victims do not always report these cases (Du Toit 2015). Even if they do report them, their cases are often not prioritised or even investigated (Sibanyoni 2018). Most South African public universities have neither preventative nor control mechanisms to curb sexual assault, let alone reporting structures when violence has been committed against female students. Moreover, student support service units are usually understaffed and the staff are not adequately trained to handle cases of sexual victimisation. For example, UKZN has a call centre where gender-based violence may be reported, but this centre is ineffective as it has limited and inexperienced staff that have not been trained to deal with the issues of sexual victimisation. This call centre is not designed to deal specifically with sexual offences as it addresses all forms of violence against women. Moreover, it is not functional 24/7 and the institutional security personnel reportedly receive no training in handling sexual victimisation cases or in assisting the SAPS to investigate such incidences (Dastile 2004).

Even though the sexual victimisation of female students has received attention from the South African government, mechanisms to curb this problem have not been implemented. For instance, as many as 50 rape cases were reported in 2018 across 26 South African universities (Nkosi 2018). This figure does not include the many sexual victims who refrained from reporting rape. Sibanyoni (2018) argues that this lack of reporting is exacerbated by a lengthy, time consuming, and degrading process.

Sexual violence against women and children appears to be more common in settings where gender roles are rigidly enforced and where masculinity is associated with toughness and dominance whilst femininity is associated with submissiveness. The South African society is still infused with dominating perceptions of patriarchy, superior masculinity, and unequal gender roles where men dominate and women are the ‘inferior gender.’ These perceptions perpetuate the victimisation of women in many ways, particularly sexual abuse, and universities are unfortunately not exempt from this phenomenon (UNAIDS 2016).

### Theoretical framework

The following discussion pertains to the theoretical framework underpinning this study.

#### An integrated model of sexual harassment and rape on campus

**Fig. 1:**
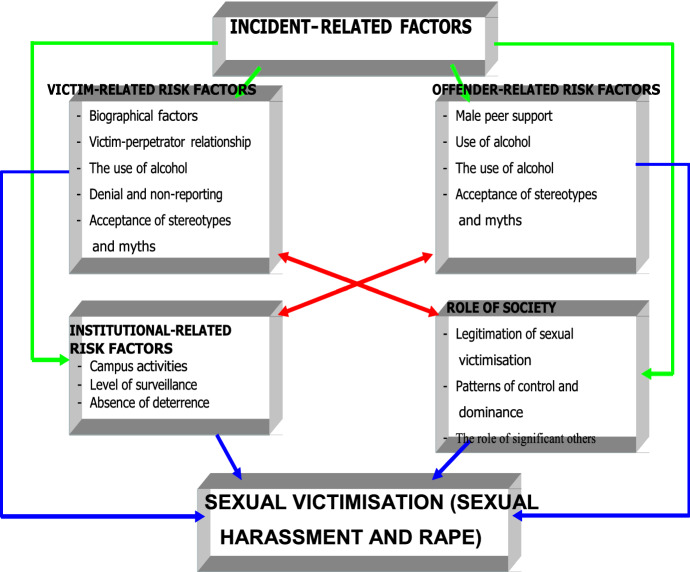
Sexual victimisation (sexual harassment and rape)

A model to address the occurrence of rape on campuses was developed by Dastile (2004). The point of departure of this model is that the convergence in time and space between the motivated offender and the potential victim in the absence of a capable guardian or guardians provides the opportunity for sexual harassment and, more direly, the rape of female students on the campuses of tertiary institutions. The model assumes that various victim-related and offender-related risk factors, institutional risk factors, as well as societal risk factors interact to encourage the sexual harassment and rape of female students on campus. This model was used to underpin the current study, as it provides a comprehensive explanation of the phenomenon under study. For the purpose of this paper, certain less relevant risk factors will be omitted in the effort to focus on only those factors that are applicable to the sexual victimisation of female students in the three selected KwaZulu-Natal institutions (Fig. 1).

### Risk factors that contribute to the sexual abuse of female students

#### Victim-related risk factors

According to Dastile (2004: 2), the sexual harassment and rape of female students in tertiary institutions are attributed to a number of risk factors. These include biographical factors, the victim–perpetrator relationship, the use of alcohol, denial, non-reporting, and stereotyping. Each of these will be discussed in detail to elucidate how they facilitate the sexual victimisation of vulnerable female students at tertiary institutions.

#### Biographical factors

Demographic variables such as age and gender play an important role in the sexual victimisation of young people (Ageton 1983: 34; Clark and Lewis 1977: 58; cited in Dastile 2004: 3). It is particularly females between the ages of 18 and 25 who are at high risk of sexual harassment and rape as they are often seen as vulnerable and therefore suitable targets for sexual perpetrators. Such young and naïve young girls are often ready for ‘a good time’ and are therefore prone to forming close relations with men of the same age group (Dastile 2004). Hindelang, Gottfredson, & Garofalo (1978) state that the risk of victimisation depends on the extent to which victims and offenders share the same demographic characteristics (Hindelang, Gottfredson, & Garofalo, 1978), which is a notion that Paludi (1996: 112) and Sandler and Shoop (1997: 110) share when they state that first-year female students are at high risk of sexual harassment and rape on campuses. These students, who only recently graduated from high school, know very little about university life and many are highly susceptible to the peer pressure and new-found freedom they enjoy away from the familial home.

#### Victim–perpetrator relationship

According to Dastile (2004), the sexual harassment and rape of female students at tertiary institutions depend on the relationship between the victim and the perpetrator, as most incidences of rape on campuses occur between men and women who know each other (Bohmer and Parrot 1993: 20; Russo 2000: 2; Dastile 2004: 4; Sandler and Shoop 1997: 14). This is in direct contrast with the generally accepted belief that rape occurs when the perpetrator is a total stranger to the victim (Bohmer and Parrot 1993: 20). Moreover, the perpetrators of date and acquaintance rape tend not to define their act as rape as they argue that the relationship that existed prior to the offence was mutual. Moreover, the victims of such rape incidences rarely suffer any severe physical bruises or scars because of limited physical force and it therefore becomes very difficult to prove an incident of rape in such cases. The offender often manipulates the victim to submit to sexual intercourse, and this makes it difficult for the victim to report the case as it is a matter of his word against hers.

Sexual harassment on campus is mostly perpetrated by a staff member against a student or by a student against another student (which is also referred to as peer harassment). The former represents *quid pro quo* harassment which is perpetrated by an individual who has more power over the other, whereas peer harassment occurs amongst students who are classmates or acquaintances (Dastile 2004: 4).

#### Alcohol abuse

Many acts of violence occur whilst persons are under the influence of alcohol or other substances. Too much alcohol intake can impair the victim’s ability to clearly communicate her rejection of sexual advances and this makes her vulnerable to sexual harassment and rape. Moreover, if a victim was under the influence of alcohol during the incident, she might be unable to account for the event later (Muehlernahrd and Linton 1987: 186) and her version of the rape may then not be believed (Unit for Gender Research in Law at Unisa 1998: 105). The stereotyping of female students who use alcohol, particularly when they do so in excess, is common on campuses. For instance, they are labelled as promiscuous, sexually available, and ‘easy’, and this makes them vulnerable to rape and sexual harassment. In severe cases, insensitive prowling male students argue that females who drink are not worthy of respect and thus deserve to be ‘punished’ (Kanin 1985: 224, cited in Dastile 2004). Some males even argue that such females ‘ask for it’.

Female students who attend parties and then drink to excess are obviously at risk of sexual harassment and rape. Such females’ friendliness and apparent lack of inhibition when they are under the influence of alcohol are perceived as an invitation for sexual activity (Dastile 2004), regardless of the fact that they might refuse sexual intercourse. Being intoxicated compromises females’ rational thinking and makes their position vulnerable and precarious in the presence of equally intoxicated males. Many instances of harassment and rape thus occur during a ‘braai’, a ‘bash’, or even sporting and musical events when alcohol is freely used on university premises. Alcohol is also freely used in residences and during parties that are hosted on weekends. Prowling males use such opportunities to get a female target drunk with the intention of raping her, whilst some perpetrators use alcohol to give them the courage to victimise vulnerable girls.

Dastile (2004) is of the view that the consumption of alcohol is one of the most predominant causes of sexual harassment and rape at tertiary institutions. Being under the influence of alcohol is used by perpetrators to rationalise their behaviour, reduce personal responsibility, and present a socially acceptable excuse to engage in otherwise prohibited behaviour (Dastile 2004: 7). When rape occurs when the male is intoxicated, the perpetrator’s actions may be seen as justified by others. He uses the excuse, which is often accepted as ‘diminished responsibility’, that he was under the influence of alcohol and does not remember anything. He thus shifts the blame and refuses to accept responsibility for his actions.

#### Offender-related risk factor

The identification of offender-related risk factors provides some answers as to why certain individuals choose to sexually victimise females who are defenceless and vulnerable. These offender-related risk factors include male peer support, the use of alcohol, and the notion that certain stereotypes are available for abuse.

#### Peer support for males

Dastile (2004) argues that various student groupings are found on campuses such as sport enthusiasts or other groups that are formed due to a common interest or cause. The key to the existence of these groups is that students desire to experience a sense of belonging and identification. However, participation in such groups carries some risk of sexual victimisation. Many male students experience stressful dating relationships due to sexual frustration and challenges associated with patriarchal authority. Some male students try to deal with these problems on their own, whereas others turn to friends for guidance and support. However, the advice they receive may encourage them as it justifies sexual harassment and even rape. Some types of social support thus have negative consequences and endanger the safety of female students who are in dating relationships at university (Dastile 2004).

#### Institutional risk-related factors

Institutional risk-related factors that encourage sexual harassment and rape are associated with the campus environment. These factors play a significant role in facilitating or preventing sexual victimisation at institutions of higher learning.

#### Campus activities

A campus is a relatively open and free space where not only academic but also social and physical activities are encouraged for the holistic development of students. Although demarcated and equipped with security gates and entrance control measures, most campuses are not closed to the public as it is considered inappropriate to do so. As institutions that render a service to society, campus boundaries are often not firm and many buildings are kept open for public use. Universities thus also attract people from surrounding communities. It is thus difficult, especially if there is no tight access control in and out of campuses and residences, to keep track of who is on campus for academic or for criminal purposes (Dastile 2004).

The campus community also engages in a wide range of societal activities, whilst ethnic customs, cultural norms, and family histories underpin the behaviour and activities of campus dwellers. Sadly, some of these habits, customs, and norms encourage and condone the abuse of others (Dornhoff 1983: 143; Johnson and Sigler 1997: 55), and such behaviours often contribute to some becoming the perpetrators of violence whilst others fall victim to acts of abuse and torment. Bars on or near campuses and other activities such as parties where alcohol is commonly used and abused open the door widely for the victimisation of those who are vulnerable, especially young females. These factors make campuses attractive spaces for motivated offenders (Dastile 2004).

#### Poor surveillance

According to Dastile (2004), social characteristics associated with sexual harassment and rape on campus include access to campuses and easy escape routes, deserted and isolated areas, poor surveillance and lighting, and poor security measures, such as cameras on campus. An area that is not well lit and is isolated, especially after dark, is highly likely to be seen as an attractive target area, especially if female students have to walk across such areas to reach a residence, bus stop, or taxi rank. These females are highly at risk of harassment, especially if they walk singly or in small groups.

The number of pedestrians in some areas on campus is also a factor that may encourage attacks. For instance, students who attend classes at night or study in the library and walk to a residence or taxi rank after dark are suitable targets for attack. When students leave after evening classes they tend to walk in groups, but as they disperse the number in the group gradually diminishes. It is thus not uncommon for a female student to walk alone at night as she heads towards her dormitory; a motivated offender may thus follow her and subject her to sexual harassment and even rape. Such incidents occur after dark when fewer individuals are on campus who can distract a motivated offender (Dastile 2004).

#### Absence of deterrents

According to Dekeseredy and Schwartz (1997: 131), the perpetrators of sexual victimisation on campuses are rarely sanctioned as their victims are often unwilling to report the incident to the authorities due to a number of reasons. Firstly, university authorities are reportedly quite reluctant to take action against such offenders because they fear negative publicity that will tarnish the image of the university (Fisher & Sloan, 1988:167). Secondly, some perpetrators occupy special positions in the university hierarchy, such as being members of the SRC or the university disciplinary committee. Cases of appeal to university authorities in charge of a case are thus often dismissed or the sanctions are overturned or reduced. Thirdly, a blind eye is often turned to sexual harassment due to a lack of understanding on the part of the university community (administrative personnel, academic staff, service staff, and the student population at large) of what constitutes rape (especially date or acquaintance rape) and sexual harassment (Dekeseredy and Schwartz 1997: 5; Fisher et al. 2000: 12). It is for these reasons that most victims do not characterise the sexual victimisation they experienced as a crime, and this deters them from reporting such cases.

## Material and methods

This study adopted a qualitative research approach to identify and explore the causative factors of sexual harassment as well as the predispositions, perceptions, assumptions, and experiences related to the sexual victimisation of female students. According to Burns (2000), qualitative research is a valuable approach for exploring and understanding a social phenomenon, which in this study was the sexual victimization of female students.

### Sampling

The study sampled a total of 60 participants using purposive sampling to select the most relevant and knowledgeable participants whose views would contribute to achieving the objectives of the study. The sample comprised 30 students, 15 risk management staff (RMS) members, and 15 student support staff members. The researchers purposively sampled 10 students from each of the three universities, five RMS staff members from each university and five student support staff members from each university. These participants were deemed key informants because it was envisaged that they would possess first-hand information on the issue under study as they had both knowledge and experience of students’ experiences of sexual harassment on their respective campuses. The participants had to be registered university students or be employed in the RMS or Student Support Services unit and be between the ages of 18 and 50. Only staff members who had at least two years’ of work experience were selected. Socio-economic background, race, and gender were not inclusion criteria. The students who were included had to reside in on-campus accommodation as such students generally spend a lot more time using campus facilities compared to those who arrive during the day and leave again after classes. The RMS members were selected as they would have first-hand information on the topic as they are the first point of contact for harassment victims, whilst Student Support Service staff members were selected as they are the secondary support structure who counsel victimised students. The study did not only recruit victims of sexual violence, but also people who would have knowledge of such incidences. All ethical considerations were adhered to in the sampling and interview processes.

Due to the COVID-19 pandemic that broke out in March 2020, the sampling of participants had to be amended. A few participants were purposively sampled face-to-face but, due to restrictions, some participants were recruited by approaching HoDs by email or telephone to request access to their staff. This technique was highly effective as the HoDs shared the contact details of staff members who had been approached and declared their willingness to participate. The researcher communicated with these staff members and explained the purpose of the study. Zoom interviews were conducted. The study received full ethical clearance from the Research Ethics Committee of the university, whilst gatekeepers’ approval letters were received from all three selected institutions.

### Study sites

University A was established on 1 January 2004 when two established universities merged. It has five campuses with a total student population of over 45 000 and an academic staff complement of 1 348. (UNIPAGE, 2020).

With approximately 30 000 students, institution B is the first choice for higher education in KwaZulu-Natal. This institution has five campuses in Durban and two in Pietermaritzburg. In July 2019, approximately 33 932 students were enrolled to study at this institution. The university is one of five technical institutions on the African continent to offer Doctoral degrees. This university shares its geographical location with institution A and is located within broader communities, hence cannot be treated in isolation from them.

Institution C is situated on the outskirts of Durban, South Africa, on a site overlooking the Indian Ocean. It accommodates 13 000 students date. It is located in the academic hub of the eThekwini Metropole which has a dynamic economy and is focused on future growth. It is a residential university that largely serves previously disadvantaged communities. It has the strategic intent to educate and empower students and communities and to address the pressing transformation and multicultural issues facing South Africa (Institution C webpage).

Owing to the sensitive nature of the investigation, the names of the institutions will not be mentioned. Instead, they are referred to as A, B and C. Suffice it to say that they have certain features in common and that they are all home to students who pursue an academic career. They are also located in the Durban area in KwaZulu-Natal and their student enrolments are dominated by black South Africans. The largest population group in the Durban area is the Zulu people, whilst about 15.3% are White, nearly one-quarter of the population is Indian or Asian and 8.6% are Coloured (Durban Population, 2022). Sixty-eight percent of the city’s residents are under 19, whilst 68% are of a working age.

### Data collection and analysis

As the nature of the investigation was sensitive, semi-structured interviews were used as a data collection tool. This technique allowed the researchers to actively engage with the participants to explore their perceptions and opinions and to elicit data that would address the research questions. It also enabled probing for more information, clarification of answers, and face-to-face contact with the participants. This technique further allowed the researchers to explain or clarify questions, thereby increasing the likelihood of eliciting valuable responses from the participants. The interviews were conducted in tranquil and private venues and an audio recorder was used, as proposed by Bless, Higson-Smith, & Sithole (2013). The participants were encouraged to talk freely about their experiences and were then probed for more in-depth information as fresh topics emerged. Each interview took approximately an hour to complete. The researchers rigorously adhered to all ethical considerations during the sampling and data collection processes. The interview schedule is attached as Appendix A. The participants, who signed a consent form before the interviews commenced, volunteered to be part of the study and could withdraw at any stage. The collected data were analysed thematically.

## Results and discussion

The participants are identified according to their institution as either A, B or C. Codes are allocated as follows:

S = student and number of participant,

SS = security staff member and number of participant, and

SC = student counsellor and number of participant.

Insert: A table summarising the characteristics of each subsample (students, risk management staff and student support staff) is attached as Appendix B.

### Factors that contribute to the sexual victimisation of female students

Excessive alcohol use was highlighted as a pivotal factor in incidences of sexual victimisation. The participants agreed that the student culture of alcohol consumption exacerbated the vulnerability of female students. One participant commented as follows in this regard:

“The information that young girls go out drinking and sometimes with strangers is a cause of vulnerability. I would say that alcohol consumption is the biggest factor that causes female students to be victimised. They have this culture in residences of inviting each other for movies or parties where there is alcohol with the intention to sleep with these females. At the end of the day it is sexual victimisation because the female does not consent to this and was not there for sexual intercourse from the beginning. Alcohol plays a huge role in these incidences happening” (A: S3).

Another factor that exacerbates the vulnerability of female students is the use of drugs, which was highlighted as follows:

“I have stayed in campus residences for quite some time now and many of them are victimised when they are either high on a certain drug or intoxicated with alcohol. I would say they become vulnerable and easy targets when they are under the influence of alcohol. There have, however, been cases of sober victims and perpetrators. It could be those in intimate relationships” (C: S6).

A security staff member also referred to situations that are conducive for victimisation:

“Alcohol consumption is the main contributing factor. It is very unfortunate because we don‘t allow students to bring alcohol onto campuses or in residences but they will go and drink it then come and cause trouble. Students also participate heavily in night lifestyles where they invite each other to attend parties where alcohol is involved. We then get reports after those parties that a certain student was victimised” (B: SS1).

Another security member raised the issue of both the victim and perpetrator being intoxicated:

“Definitely alcohol consumption. Students always have money from NSFAS^1^ , so they can always afford alcohol. They drink and then victimise each other. Males will take advantage of a drunk female or sometimes a male will drink alcohol and then can‘t control himself. This results in inappropriate behaviours with other students. Sometimes both the victim and perpetrator are drunk during the time of the incident” (B: SS5).

From the narratives above it can be inferred that although institutions of higher learning have strict rules that prohibit the consumption of alcohol on campus, student culture has evolved to the extent that such regulations are ignored. A study by Umana et al. (2014) suggests that about fifty per cent of college students engage in binge drinking. The later authors argue that the use of alcohol exacerbates promiscuous sexual activity and reduces the ability to avoid violence. Monks, Tomako, Palacios, and Thompson (2010) agree and argue that the physiological effects of alcohol, such as the disruption of higher-order cognitive processes, increase the risk of assault. Alcohol alters perception, decreases reaction time, and impairs decision-making. When used excessively, it alters the cognition ability and autonomy of an individual, hence intoxicated students either become perpetrators or victims of sexual abuse. Langton and Sinozich (2014) found that most incidences of sexual misconduct on campus started when individuals who were known to each other met and started drinking, but at some point that consensual contact turned nonconsensual, resulting in sexual violence.

### Effects of sexual victimisation on female students

Female student victims experience various forms of psychological harm due to sexual abuse, ranging from emotional and physical trauma to social withdrawal. Various studies have suggested that rape, or any other form of sexual victimisation, has an impact that exceeds that of other crimes. For instance, the health consequences of sexual violence include both short- and long-term health problems, such as depression, eating disorders, post-traumatic stress disorder, and suicidal ideation (Black et al. 2011; Campbell et al. 2009; Gidycz et al. 2008; Kaura and Lohman 2007). Other consequences are physical injuries, sexually transmitted infections, and chronic illnesses (Campbell et al. 2003; Fisher et al. 2000). According to Brown (2000, cited in Sibanyoni 2018: 79), mental health problems are increasingly experienced as a common consequence of sexual abuse. Young people who were exposed to sexual victimisation often exhibit symptoms of post-traumatic stress disorder, borderline personality disorder, and/or dissociative identity disorders. The short-term impact of sexual victimisation may include dropping out of university, difficulties in communication, and academic delays (Brown, 2000, cited in Sibanyoni 2018). The findings of the current study corroborated Brown’s findings, as the participants noted that affected students usually withdrew from academic life as they seldom attended classes, did not hand in assessment tasks, and avoided writing exams. Similar to earlier studies, the current study thus also found that sexual victimisation affects victims’ academic performance and that, as a result, they constantly repeat modules or drop out of university. Examples of the participants’ comments in this regard are the following:

“The victim faces a range of effects from the victimisation that was invasive in nature. These are physical, medical or STIs, and pregnancy. Other effects are self-blame and withdrawal from academic and social activities. This has long-term effects because the victim may perform poorly in his studies due to the trauma of being victimised” (A:SC1).

“This attacks a person’s self-esteem and impacts on their entire identity resulting in a loss of identity. The stigma attached to being sexually victimised causes a lot of damage in a person’s social life and well-being. The results of this also affect academic performance” (C: S2).

The immediate effects on affected students’ academic performance were explained as follows:

“The cases I have attended to have resulted in the victim performing poorly in their academics. They are sent to us for counselling but sometimes the damage is so deep that it causes students to not perform well academically” (B: SC4).

Failure becomes a reality, as an SS participant stated:

“It affects the person badly. You find that the victim performs poorly and even fails exams. It has long-term effects; a person will suffer for a long time” (A: SS1).

As stated by Holland and Cortina (2017), experiencing sexual assault can have devastating consequences for survivors’ psychological and educational well-being, which may intensify if survivors do not receive adequate care. This study thus confirmed that sexual victimisation adversely affects victims’ academic performance.

It is also a fact that students who were sexually violated experience various psychological effects, such as depression, PTSD, STIs, HIV, unwanted pregnancy, and a poor self-image. Amar and Gennaro (2005) state that victims’ reports of violence and associated changes in routines and behaviours resulted in poor class attendance and, ultimately, in academic failure. This could be attributed to depression and feelings of anxiety that diminish a female student’s commitment to academic work and even diminish her ability to engage with other students due to social blame, shame, and embarrassment (Jordan et al. 2014).

The study also found that students who had experienced sexual victimisation in the universities under study experienced long-term effects, such as post-traumatic stress disorder, depression, acute fear and anxiety, generalised anxiety, suicidality shock, and social withdrawal. These findings corroborate those of Herman (1992), Campbell et al. (2009), and Jordan et al. (2010). For instance, a participant stated that concomitant effects of sexual trauma are stress and depression. This was explained as follows:

“It [sexual trauma] alters the person’s self-confidence because one starts to see themselves the way that the perpetrator has put it. For example, if something is said about one’s dress code or appearance, then the victim will develop negative feelings about theirself. It also evokes feelings of trauma and lack of trust towards men. They fall into a phase of depression” (B: S5).

The onset of depression and its devastating effects on the well-being of the victim were shared by a student participant:

“Sexual victimisation impacts very negatively on a victim regardless of gender. It puts the victim in a mental disequilibrium because now they are put at a crossroad whereby they need something but they also have to give up something in return. Sometimes our needs get the better of us and then we find ourselves succumbing to those demands. It is a very difficult and uncomfortable situation and I have seen people go through it. It impacts on their self-esteem because now they have this baggage of experience knowing that someone victimised you because you were in need. I have been exposed to it but fortunately enough I was able to identify the signs and take my stand against it. However, sexual victimisation is highly prevalent in our institutions and I’m so grateful that you came up with this research because I believe that this is the starting point of addressing this issue” (A: S1).

Depression was highlighted as a pervasive effect of sexual trauma and was iterated by all the participants. One participant commented on this issue as follows:

“It affects the victim in so many ways. I would say that it hinders the way a person thinks. I can imagine one trusting someone only for that person to victimise you. It distorts the trust not just in that one person but in the entire humanity. They don’t know who to trust. The victim becomes paranoid and you start blaming yourself and falling into depression. It can damage the victim physically and mentally; some will even try harming themselves just trying to forget what happened. It damages the person’s health” (B: S3).

Depression and self-harm, with long-term ramifications, were linked by some participants. One student verbalised this as follows:

“It is something that leaves long-term trauma to the extent that some victims end up having hatred towards men. Some will even have problems in their romantic relationships because the victim will not trust any man. This requires a lot of therapeutic support” (C: S1).

It was also argued that sexual trauma can recur:

“It evokes trauma. They are always reminded of what they went through” (A: SC1).

The participants also referred to severe psychological trauma due to the impact of power dynamics on female victims. A student counsellor stated:

“There are a lot of psychological effects and often the self-esteem of the victim is damaged. The victim experiences feelings of guilt, shame, and powerlessness which causes stress. Many victims that I have spoken to are very traumatised and mention that they keep getting flashbacks of the incident. They exhibit symptoms of depression which also affects their academic performance” (B: SC5).

A study that was conducted earlier at a historically black university found that sexual assault survivors experienced significantly more symptoms of depression and were more likely to screen positive for post-traumatic stress disorder (PTSD) than students who had not been victims of such assaults (Lindquist et al. 2013). The current study thus confirmed that the long-term effects of sexual trauma have dire implications, particularly when the victim healed physically but without any form of therapeutic support.

### Underreporting of sexual victimisation at tertiary institutions

Sexual victimisation is regarded as a silent epidemic due to victims’ reluctance to report these incidents (Abbey et al. 1996). This suggests that statistics are unreliable and do not reflect the true picture of the extent and impact of sexual assault on university campuses. More than half of the student participants in the current study admitted that they were not aware of where they could report an incident of sexual victimisation.

A number of factors lead to students’ unwillingness to report sexual victimisation. One factor seems to be the fact that the perpetrator may be known to the victim. Another factor is that victims feel that they will not be believed by the authorities. Sibanyoni (2018:94) states that many sexually abused victims do not disclose the act because of threats, blame, and bribery, whilst Mudaly and Goddard (2006, cited in Sibanyoni 2018:95) state that victims experience feelings of hesitancy and ambivalence concerning disclosure. According to Ullman and Najdowski (2010), victimised students fear not getting support when reporting victimisation, whilst most feel embarrassed and fear that people will not believe them, particularly if the perpetrator is a relative and not merely an acquaintance or a stranger.

Jensen (2005) points out that victims are sensitive to the reactions of those to whom they disclose the abuse and that most fear the misinterpretation of their stories. Spies (2006:48, cited in Sibanyoni 2018:96) encourages confidants who hear the confession to show responsibility and believe the victim. The findings of the current study clearly suggest that sexual violation devalues, degrades, and severely harms female student victims, especially if they are not believed. Disbelieving their confession obviously creates frustration and strain and ultimately results in poor academic achievement. Many female students tend to blame themselves for the abuse, as proposed by Killian and Brakarsh (2004), who also suggest that self-blame is a reality. Ullman and Najdowski (2010) argue that self-blame is more likely to occur in cases where the perpetrator is a relative of the victim.

One participant explained the reasons for the underreporting of sexual violence as follows:

““It is definitely our justice system; it has failed many victims before so each time a victim wants to report they first think that even if they come out about it in the end justice won’t be served. Instead people will blame them and make matters worse. They are running away from the secondary victimisation because on top of the turmoil that the victim is already faced with, after reporting they now have to deal with people blaming them for being victimised” (B: S9).

The lack of supportive institutional structures for sexual victimisation is one of the most common reasons for non-reporting. Garcia et al. (2012) state that there is a decreased likelihood of students seeking assistance on campuses where they do not trust the institution’s ability to respond. This, according to Smith and Freyd (2014), is because these students experience a sense of institutional betrayal. The participant listed below elaborates on this:

“There goes a young woman who was actually failed by all structures of the institution I believe she attempted twice to commit suicide as she didn’t perform well academically and the last time I met her she was having difficulties with registration. She was on the verge of academic exclusion, furthermore one would imagine the psychological impact it would have on her and the negative effects it will have on her reputation” (A: S7).

Institutions of higher learning are renowned to protect the perpetrator as the S participant clarified:

“Most students don’t report sexual victimisation because these kinds of incidences are normally swept under the carpet. For example, when an employee victimises a student they are only just dismissed and students are not warned that something like this happened within the institution just to raise awareness. They often protect perpetrators when the perpetrators victimise students. A follow-up and debriefing should be done so that it is brought to our attention and we are made aware of what is happened and whether justice is served. Students don’t report because they do not observe any commitment from the institution to deal with these issues of sexual victimisation of female students” (A: S1).

Few South African universities, including the three universities under study, have reporting mechanisms that allow students, parents, or caregivers to lay a complaint or charge about sexual victimisation perpetrated on a campus. Members of professional student support service units assist students who experience problems, but overburdened and unmotivated staff members cause the secondary victimisation of students by being uncaring, simply dismissing their concerns, and being overly preoccupied with following certain protocols that often humiliate the student victim (Killian and Brakarsh 2004). When students try to report their ordeal to support staff, they are often confronted with a lack of caring and empathy and support staff often blame the victim for the incident. It has been speculated that interventions following a victim’s disclosure of abuse may be even more traumatic than the actual abuse and may thus contribute to the overall traumatic experience (Berliner and Conte 1995). A participant offered the following comment in this regard:

“Students and staff members are the main perpetrators, although many of the cases are those of intimate partners. Most females are victimised by their boyfriends but don’t report it because they feel obligated to have sex even when they don’t want to. This is also the reason why some cases go unreported” (A:SS2)

In the current study, only three participants had experienced attempted sexual violence, but not rape. They referred to these incidences as ‘harassment’ and ‘verbal victimisation’. Based on the data, the researchers postulate that, even though underreporting is sometimes due to barriers that exist within the criminal justice system, the relationship between the victim and the perpetrator presents significant challenges and is often why a student is unwilling to report a case of sexual abuse to the police or anyone else. Another challenge could be that students may blame themselves for what happened to them or they may simply accept that such incidents are part of their everyday lives.

### Policing sexual victimisation at institutions of higher learning

The study found that there were a lack of security force visibility on each campus. According to the participants, the private security companies seldom patrolled the campuses and some particularly avoided security presence at on-campus residences. They argued that security personnel should maintain visibility, especially during campus events or on weekends when most acts of sexual victimisation occur. A student participant, who was one amongst those who were critical of the security strategies employed by the respective institutions, shared the following insights:

“There are fundamental questions that one must put into account regarding your question: Are the structures put into place well equipped enough to deal with these matters? Do we have enough preventative measures to curve such behaviour? Do we have qualified practitioners in place to handle these matters? Unfortunately, I would respond with a ‘No!’ to all these questions which is a sign that our university still has a long way to go with regards to dealing with sexual victimisation” (A: S7).

The issue of resources emerged in the response below:

“The university has the resources but they do not utilise the resources that they have. For instance, there are counsellors in our institution, there are trained social workers who are lecturers, and they are registered with the Board to counsel people and are legally allowed to offer counselling services. There are also RMS members who are trained to investigate cases but as much as we have those services, they don’t do follow-ups. And if you don’t pester them, then your case will become a cold case. They will tell you that they have 250 cases and make you feel as if your case is not special. I feel like if they allocate services accordingly, it will show how they treat sexual victimisation seriously. I am not saying cases of theft are not important, but sexual victimisation must be a top priority. But you find that I attend a counselling session online, on ZOOM in our days, and the counsellor won’t do a follow-up or recommend an available counsellor for whenever I might be triggered I can talk to them. They treat counselling as a side job, not a priority. So that’s why I say they have the resources but don’t use them efficiently” (C: S6).

Student counsellors and security staff members averred that the interventions in place for policing sexual victimisation were ineffective. However, below are the responses of SC and SS participants who referred to noteworthy improvements:

“Honestly, it was never too good, but recently the university has upped their approach. The university has hired a private company to assist the institution with strategies to deal with sexual harassment. Security has been beefed up and there has been an enactment of committees that are very influential. Partnerships are formed with different institutions to create a system of support. There are GBV satellite units that the institution is coming up with. Those hubs are where students can report and access assistance 24 hours a day. These spaces will be conducive for students to open up in a comfortable space with trained staff members” (A: SS1).

The participants were aware of both proactive and reactive interventions to address campus issues:

“I am aware that we have policies that promote zero tolerance for GBV. We also hold dialogues and seminars. Students who report victimisation are supported and the perpetrator is expelled and faces criminal charges” (B: SS1).

Security measures have also been upgraded:

“There has been an installation of CCTV cameras in campus facilities. There are also patrols. The institution emphasises the Code of Conduct, especially to regulate students in residences and ensure that visitors are not staying during odd hours when they are not allowed” (B: SS2).

“Security has been upgraded for access to the campus. There are also campaigns to create awareness and dialogue” (B: SC4).

The students’ and staff members’ responses were somewhat contradictory in terms of effective strategies to curb sexual violence on campus. The majority of the student participants, who felt that the institutions failed to deal with the issue of sexual violence on campus, were not aware of any strategies to address this issue. Those who had been exposed to cases of sexual victimisation, either by witnessing it or being a victim, voiced their disappointment in their respective institutions and how they handled this issue. Lindquist et al. (2016) also revealed that several students expressed dissatisfaction with campus police officers, as they perceived them as intimidating. They were also critical of counselling centres which they perceived as embarrassing or clinic like. This study exposed the same flaws as the participants felt that a lack of sexual abuse reporting we due to unfriendly and unaccommodating support staff. According to Lindquist et al. (2016), many students emphasised the need for comprehensive and free services such as crisis centres, 24/7 hotlines, women’s centres, support units that specifically focus on sexual assault, more and better trained counsellors, and health centres that are open on weekends and do not require appointments. These clearly suggest a need for a comprehensive and holistic approach to dealing with the sexual victimisation of female students.

Conversely, some student participants commended their institution for its efforts to deal with sexual and other acts of violence on campus. These respondents were predominantly from institution B, which suggests that further research should be conducted at this institution to share its model/strategic plan with institutions that struggle to ensure the safety of all their students.

In general, however, the universities under study could be regarded as unsafe for their female students, particularly as the campus environment was not deemed safe by the majority of the participants. Issues such as poor lighting, a lack of security patrols, a lack of environmental crime prevention measures, such as biometrics control at gates to monitor students and visitors’ access, and a lack of adequate security personnel at residences were highlighted. Clearly, these limitations all contribute significantly to the victimisation of female students on the campuses under study.

## Conclusion and recommendations

The study exposed the reality of a considerable number of acts of sexual violence that are committed against female students at the three selected tertiary institutions in KwaZulu-Natal. These acts were mostly perpetrated by people known to the victims. Alcohol and drug use/abuse during ‘bashes’, pub visits, and residence parties are clearly a major contributor to the sexual victimisation of female students. Perpetrators sometimes slip drugs into females’ drinks or wait until they are highly intoxicated before sexually molesting them. Many sexually violated victims experience depression, trauma, unwanted pregnancy, HIV/AIDS, STIs, withdrawal from the university’s academic activities, difficulties in communication, and/or academic delays, which means that many fail their courses. The study confirmed the reluctance of female students to report sexual victimisation to the authorities. This means that the perpetrators do not face a criminal justice process or conviction, and the message that this sends to potential sexual predators is devastatingly clear: you may select your victim and do with her as you please with impunity.

Various physical, procedural, and attitudinal barriers to reporting and recording sexual victimisation exist, and these barriers discourage female students from reporting the sexual victimisation they experienced to a designated authority. As the word of female guilt and male dominance has spread, many female students may feel that they will not be taken seriously and that they will be treated as the guilty party who ‘asked for it’ or instigated their ordeal, whether by their attire or behaviour.

The study recommends more regular patrols, CCTV surveillance cameras, and lights everywhere on university campuses. Security guards should be stationed at residences and be alert to activities of both individuals and couples. Security guards and police officers should be visible and alert at every major university event, such as fresher ‘bashes’. Security personnel must be capacitated with investigative skills to help the police as they are on campus and should know how to deal effectively with sexually violated victims before the case is referred to the police.

Psychology departments at universities should play a pivotal role in facilitating counselling for sexually abused students. Clinics must be resuscitated as one-stop crisis centres where professional psychologists provide therapeutic interventions as part of a community engagement initiative. Comprehensive measures should also be implemented by university management teams to address many risk factors that contribute to on-campus sexual violence.

It is evident that sexual victimisation is a phenomenon of concern across local and international university campuses. Even though there are initiatives to fight this crime, there are still gaps in the implementation of interventions. This calls for a focus on the factors that were highlighted in this paper so that institutions are able to provide a safe teaching and learning space for tertiary students.

The limitations of the study should be noted. Firstly, as the sample size was limited, the findings may not be generalised. Secondly, some participants were reluctant to fully disclose the information they possessed, which may have compromised the data somewhat. Thirdly, many identified victims of sexual abuse declined participation. Moreover, some recruited participants withdrew during the data collection process because they found it difficult to talk about their experience as it had caused them considerable trauma.

## Supplementary Information

Below is the link to the electronic supplementary material.

Supplementary file1 (DOCX 74 KB)

## Data Availability

The data pertaining to the concluded research study can be obtained from the University of KwaZulu Natal research institutional archives. The full research thesis can be accessed via the institutions library page.

## Footnotes

1 National Student Financial Aid Scheme.
