# Supplementary material for: Female students as victims of sexual abuse at institutions of higher learning: insights from Kwazulu-natal, South Africa
Source: SN Soc Sci. 2023 Feb 11;3(2):40. doi: 10.1007/s43545-023-00611-z (PMC9919736; doi:10.1007/s43545-023-00611-z)
Supplement: Supplementary file 1 — Supplementary file1 (DOCX 74 KB) [file 43545_2023_611_MOESM1_ESM.docx]

**APPENDIX 1: INTERVIEW SCHEDULE**


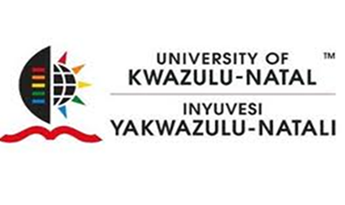
**Interview schedule**

1.How long have you been a student/staff member at this institution?

**Nature:**

2. How would you define sexual victimization of female students in this context?

3. Please highlight a few examples of what constitutes as sexual victimization in your institution?

4. What is the nature and serious of sexual victimization incidences on this institution?

5. Does it frequently occur at night or during the day?

6. These incidences. Where on the parts of the institutions do they happen?

**Contributing factors:**

7. What would you say are the contributing factors to female student sexual victimization?

8. Do these factors vary between male and female students?

9. Would you consider the geographical location of the institution to be a contributing factor? If yes, how so?

10. Who, in your opinion are the perpetrators of sexual victimization in your institution?

**Effects:**

11. How does sexual victimization affect the victim?

12. What effect does it have on the general student and staff community of this institution?

13. Does the institution have adequate support for the victims of sexual victimization?

**Underreporting:**

14: Are you aware of where you/ your students can report a sexual victimization incidence should you/they become a victim while on campus?

15. Studies have revealed that most victims don’t report sexual victimization incidences. Why do you think this is the case?

16. Does the institution create awareness on the steps to take should you/your students be a victim or witness of a sexual victimization act?

**Current strategies:**

17. Do you know of any current strategies adopted by this institution for policing sexual victimization of female students? If yes, please elaborate.

18. In your opinion are these strategies effective?

19. What recommendations would you give with regards to dealing with this sexual victimization of female student’s problem?

Thank you.

**APPENDIX 2:**

**SAMPLE DESCRIPTION FOR THE THREE SELECTED INSTITUTIONS.**

Below is a representation of the subsamples of each institution selected in the study. The interview schedule only had a question that asks whether a participant is a staff or student and the number of years they have been with the university. Gender was not a factor in the data collection; however, the consent letters show the gender.

There were limitations experienced during the study, where sampling is concerned. Throughout the study, the researcher attempted several times to gain access to student support services and security services for institution C to participate. Unfortunately, these two cohorts were inaccessible, and all efforts to engage them were futile. As a result, the sample size was reduced by ten (10) people. The researcher believed in voluntary participation and thus understood that if participants showed no interest in participating, they could not be included in the study.

The sample was further reduced by four (4) participants because Institution A only had one (1) student counsellor on campus who worked with victims of sexual abuse. For this study, the researcher used one (1) student counsellor. The total interviewed participants were a total of 46 participants.

| **INSTITUTION A** | | |
| --- | --- | --- |
| **STUDENTS** | **GENDER** | **YEARS IN INSTITUTION** |
| 1. Student Participant 1 | Female | 5 Years |
| 1. Student Participant 2 | Female | 3 Years |
| 1. Student Participant 3 | Female | 4 Years |
| 1. Student Participant 4 | Female | 2 Years |
| 1. Student Participant 5 | Male | 3 Years |
| 1. Student Participant 6 | Female | 3 Years |
| 1. Student Participant 7 | Male | 5 Years |
| 1. Student Participant 8 | Female | 6 Years |
| 1. Student Participant 9 | Female | 5 Years |
| 1. Student Participant 10 | Male | 4 Years |
| **STUDENT COUNSELLORS** | | |
| 1. Student Counsellor 1 | Female | 3 Years |
| **SECURITY RISK MANAGEMENT SERVICES** | | |
| 1. Security staff 1 | Female | 7 Years |
| 1. Security staff 2 | Female | 10 Years |
| 1. Security Staff 3 | Female | 3 Years |
| 1. Security Staff 4 | Female | 10 Years |
| 1. Security staff 5 | Male | 8 Years |

| **INSTITUTION B** | | |
| --- | --- | --- |
| **STUDENTS** | **GENDER** | **YEARS IN INSTITUTION** |
| 1. Student Participant 1 | Female | 3 Years |
| 1. Student Participant 2 | Male | 3 Years |
| 1. Student Participant 3 | Male | 3 Years |
| 1. Student Participant 4 | Female | 5 Years |
| 1. Student Participant 5 | Male | 3 Years |
| 1. Student Participant 6 | Female | 6 Years |
| 1. Student Participant 7 | Male | 3 Years |
| 1. Student Participant 8 | Male | 5 Years |
| 1. Student Participant 9 | Female | 4 Years |
| 1. Student Participant 10 | Male | 4 Years |
| **STUDENT COUNSELLORS** | | |
| 1. Student Counsellor 1 | Female | 5 Years |
| 1. Student Counsellor 2 | Female | 2 Years |
| 1. Student Counsellor 3 | Female | 2 Years |
| 1. Student Counsellor 4 | Female | 4 Years |
| 1. Student Counsellor 5 | Female | 2 Years |
| **SECURITY RISK MANAGEMENT SERVICES** | | |
| 1. Security staff 1 | Male | 3 Years |
| 1. Security staff 2 | Male | 2 Years |
| 1. Security Staff 3 | Male | 2 Years |
| 1. Security Staff 4 | Female | 3 Years |
| 1. Security staff 5 | Male | 2 Years |

| **INSTITUTION C** | | |
| --- | --- | --- |
| **STUDENTS** | **GENDER** | **YEARS IN INSTITUTION** |
| 1. Student Participant 1 | Female | 4 Years |
| 1. Student Participant 2 | Female | 3 Years |
| 1. Student Participant 3 | Male | 3 Years |
| 1. Student Participant 4 | Female | 5 Years |
| 1. Student Participant 5 | Female | 4 Years |
| 1. Student Participant 6 | Female | 3 Years |
| 1. Student Participant 7 | Female | 4Years |
| 1. Student Participant 8 | Male | 5 Years |
| 1. Student Participant 9 | Female | 5 Years |
| 1. Student Participant 10 | Female | 3 Years |
| **STUDENT COUNSELLORS** | | |
| **NO PARTICIPATION FROM STUDENT COUNSELLORS FOR INSTITUTION C. ATTEMPTS WERE MADE FOR THE DUARTION OF THE STUDY TO GET ACCESS TO THEM WITH NO FEEDBACK.** | | |
| **SECURITY RISK MANAGEMENT SERVICES** | | |
| **NO PARTICIPATION FROM SECURITY RISK MANAGEMENT SERVICES FOR INSTITUTION C. ATTEMPTS WERE MADE FOR THE DUARTION OF THE STUDY TO GET ACCESS TO THEM WITH NO FEEDBACK.** | | |
